# Inactivation of genes TEC1 and EFG1 in *Candida albicans* influences extracellular matrix composition and biofilm morphology

**DOI:** 10.1080/20002297.2017.1385372

**Published:** 2017-10-17

**Authors:** Beatriz Helena Dias Panariello, Marlise I. Klein, Ana Claudia Pavarina, Simone Duarte

**Affiliations:** ^a^ Department of Dental Materials and Prosthodontics, São Paulo State University (UNESP), School of Dentistry, Araraquara, Brazil; ^b^ Department of Cariology, Operative Dentistry and Dental Public Health, Indiana University School of Dentistry, Indianapolis, IN, USA

**Keywords:** *Candida albicans*, biofilm, EFG1, TEC1, extracellular matrix

## Abstract

**Background**: Infections caused by *Candida* spp. have been associated with formation of a biofilm, i.e. a complex microstructure of cells adhering to a surface and embedded within an extracellular matrix (ECM).

**Methods**: The ECMs of a wild-type (WT, SN425) and two *Candida albicans* mutant strains, Δ/Δ tec1 (CJN2330) and Δ/Δ efg1 (CJN2302), were evaluated. Colony-forming units (cfu), total biomass (mg), water-soluble polysaccharides (WSPs), alkali-soluble polysaccharides (ASPs), proteins (insoluble part of biofilms and matrix proteins), and extracellular DNA (eDNA) were quantified. Variable-pressure scanning electron microscopy and confocal scanning laser microscopy were performed. The biovolume (μm^3^/μm^2^) and maximum thickness (μm) of the biofilms were quantified using COMSTAT2.

**Results**: ASP content was highest in WT (mean ± SD: 74.5 ± 22.0 µg), followed by Δ/Δ tec1 (44.0 ± 24.1 µg) and Δ/Δ efg1 (14.7 ± 5.0 µg). The protein correlated with ASPs (*r* = 0.666) and with matrix proteins (*r* = 0.670) in the WT strain. The population in Δ/Δ efg1 correlated with the protein (*r* = 0.734) and its biofilms exhibited the lowest biomass and biovolume, and maximum thickness. In Δ/Δ tec1, ASP correlated with eDNA (*r* = 0.678).

**Conclusion**: ASP production may be linked to *C. albicans* cell filamentous morphology.

## Introduction

The members of the genus *Candida* are opportunistic fungi that are present in the oral cavity. When there is an imbalance in the immune system of the host, the oral microbiota is altered and these microorganisms may invade the oral tissues [1,2]. Clinical manifestations of infections caused by *Candida* spp. can be superficial, such as oropharyngeal candidiasis (OPC) and/or systemic candidiasis (e.g. candidemia). OPC is an indicator of the development of acquired immune deficiency syndrome (AIDS) and depending on the stage of the infection by human immunodeficiency virus (HIV), about 90% of the patients have OPC [3,4]. *Candida albicans* is the most prevalent species related to this infection [5–8].

Infections caused by *Candida* spp. are often associated with biofilm formation. A biofilm is a complex microstructure of cells adhering to a surface and embedded within an extracellular matrix (ECM), made up of secreted microbial and host-derived substances (i.e. saliva components) and products of cell lysis [9]. The ECM contributes to the build-up of the biofilm, preservation of the biofilm’s architecture, and maintenance of stable interactions between cells and between the cell surface and the environment [10]. Among the substances found in the ECM are polysaccharides, proteins, and nucleic acids, all of which play a major role in biofilms [9]. Three classes of conventional antifungals are used for treating infections caused by *Candida*: azoles (e.g. fluconazole), polyenes (e.g. amphotericin B), and echinocandins (e.g. casponfungin) [11]. However, antifungal resistance can arise from all drug classes and includes acquired resistance in susceptible strains and selection of innately less susceptible species [12].

Antifungal resistance in *Candida* biofilms is multifactorial and is associated with the physiological state of the cells, the activation of drug efflux pumps, and the protective effect of the ECM performed by β-glucans [alkali-soluble polysaccharides (ASPs)], which bind to fluconazole [13] and amphotericin B, preventing the penetration of drugs into the biofilm [14]. In addition to the protective effects of the ECM due to β-glucan, it has been shown that the extracellular DNA (eDNA) is another key component contributing to the structural integrity of *C. albicans* biofilms [15]. The ECM of *C. albicans* strain K1 was tested using *in vitro* and animal models, and the ECM composition was 55% protein, 25% carbohydrate, 15% lipid, and 5% nucleic acid, while β-1,3-glucan comprised only a small portion of the total matrix [16].

The ‘hyphal development pathway is critical for formation of significant biofilm mass’ [17]. Mutants defective in the enhanced filamentous growth transcriptional factor (EFG1), a major activator of hyphal development, presented impaired formation of a monolayer of cells on polystyrene surfaces [17]. This defect in biofilm development may occur because of altered surface-protein composition and adherence properties of the EFG1 null mutant (Δ/Δ efg1) [18]. In addition, the lack of functioning EFG1 in *C. albicans* strains yielded only pseudohyphae on solid media and without growth in liquid media [19]. Tec1p is a TEA/ATTS transcription factor which is required for hyphal formation [20]. Biofilm produced by the tec1 null mutant (Δ/Δ tec1) strain was rudimentary, less than 20 µm deep, and composed exclusively of yeast cells [17], while its parental strain formed a biofilm 250–450 µm deep that included many hyphal filaments [17]. Therefore, mutant strains defective in the filamentation genes EFG1 and TEC1 are considered less virulent than their wild-type counterparts, because they present decreased levels of infectivity of endothelial cells and plasma-coated catheters [21,22].

The study of *C. albicans* mutants with defective capability of forming biofilms is needed to evaluate the differences in the ECM components and structure when there is deficient formation of biofilm, facilitating the understanding of which components are related to a regular biofilm formation. Knowing the assembly principles of the matrix helps in deciding which component or components to focus on when designing effective new treatments to control fungal biofilm formation and pathogenesis. Therefore, the aim of this study was to characterize the ECM of wild-type (WT) and mutant (Δ/Δ efg1 and Δ/Δ tec1) *C. albicans* strains.

## Materials and methods

### Biofilm formation and processing

The microorganisms used for this experiment were *C. albicans* SN425 (WT strain), *C. albicans* CJN2302, and *C. albicans* CJN2330; the last two are mutant strains with deficient biofilm formation ability, Δ/Δ tec1 and Δ/Δ efg1 [23]. TEC1 is primarily an activator of its biofilm-relevant direct target genes and EFG1 is both an activator and a repressor [23]. The microorganisms stored at −80°C were seeded on to Petri dishes with Sabouraud dextrose agar (SDA) culture medium supplemented with chloramphenicol and incubated at 37°C for 48 h. Next, starter cultures containing about five colonies were grown using yeast nitrogen base (YNB) medium (Difco, Detroit, MI, USA) supplemented with 100 mM glucose, and incubated at 37°C. After 16 h of incubation, the starter cultures were diluted with fresh YNB medium supplemented with 100 mM glucose (1:20 dilution). These inoculum cultures were incubated at 37°C until the three strains reached the mid-log growth phase (8 h) (supplementary Figure S1). Then, the OD_540nm_ of the inoculums was adjusted to reach 10^7^ cells mL^−1^. Next, 1 mL of the inoculum of each strain was added to the wells of a 24-well polystyrene plate (Techno Plastic Products, Trasadingen, Switzerland). The culture plate was incubated at 37°C for cell adhesion to the substrate. After 90 min, the wells were washed twice with sterile 0.89% NaCl solution to remove non-adherent cells. Next, 1 mL of RPMI 1640 buffered with 3-(*N*-morpholino)propanesulfonic acid (MOPS) (Sigma-Aldrich, St. Louis, MO, USA) at pH 7 was added to each well.

**Figure 1.**
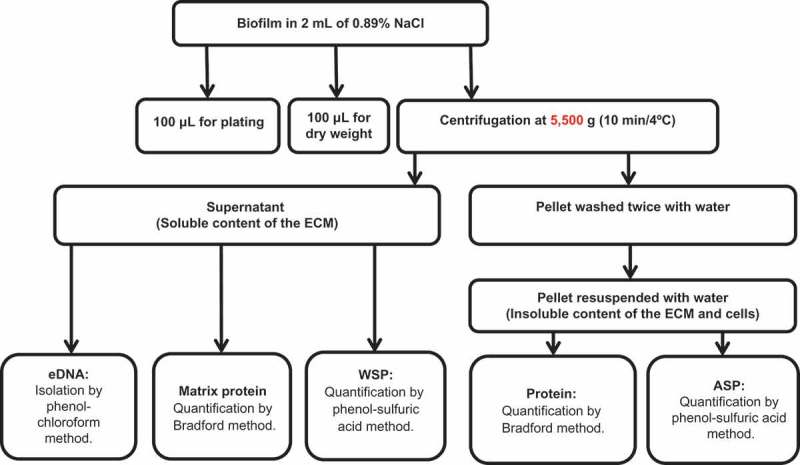
Biofilm characterization flowchart. ECM, extracellular matrix; eDNA, extracellular DNA; WSP, water-soluble polysaccharide; ASP, alkali-soluble polysaccharide.

After 24 h of biofilm formation, the culture medium was removed by aspiration and fresh RPMI buffered with MOPS (1 mL, pH 7.0) was added to each well. After 48 h of biofilm formation, the wells were washed twice with 0.89% sterile NaCl solution. Biofilms were processed following the flowchart in Figure 1. In brief, biofilms were removed by scraping each well with a pipette tip and 2 mL of sterile 0.89% NaCl. From the biofilm suspension, 0.1 mL was used for the 10-fold serial dilution and culture on SDA plates for recovery of colony-forming units (cfu) and 0.1 mL was used for dry weight (biomass) determination [24]. The remainder of the volume was vortexed vigorously at high speed for 1 min for all samples during processing for mechanical disruption of the ECM and centrifuged at 5,500 × *g* for 10 min at 4°C. The supernatant was stored in another tube and the precipitate with the cells and the insoluble components of the ECM was washed twice with sterile milli-Q water (5,500 × *g*, 10 min at 4°C). From the stored supernatant, 1 mL was separated for the quantification of water-soluble polysaccharides (WSPs) [25], 0.650 mL for eDNA analysis [26], and 0.150 mL for protein tests [27]. The precipitate was resuspended in water, then 0.05 mL was separated for the quantification of protein [27] and 0.95 mL was separated for the determination of ASPs [25].

#### Protein quantification

Proteins in the ECM (soluble or supernatant) and in the insoluble part of biofilms were quantified. The proteins from the insoluble part were extracted by boiling at 100°C for 1 h at 1,000 rpm. Bovine serum albumin solution (P5369; Sigma-Aldrich, St. Louis, MO, USA) was prepared in saline buffer and the following concentrations were used as a standard curve: 0, 0.03125, 0.062, 0.125, 0.2, 0.5, 1, and 1.4 mg mL^−1^. In 96-well plates, 200 µL of Bradford reagent (B6916; Sigma-Aldrich) was mixed with 5 µL of each curve point and biofilm samples. The reaction was carried out for 30 min, and the absorbance at 595 nm was determined in a spectrophotometer.

#### WSP analysis

An aliquot of 1 mL per sample of the homogenized supernatant was transferred to sterile centrifuge tubes to which 2.5 volumes of 95% ethanol were added. The WSPs were precipitated for 18 h at −20°C and centrifuged at 9,500 × *g* for 20 min at 4°C. After centrifugation, the supernatants were discarded. The samples were washed three times with ice-cold 75% ethanol and the pellets were air-dried. Each pellet was resuspended with 1 mL of water, and total carbohydrates were quantified using the phenol–sulfuric acid method [25]. Glucose was used for the standard curve (0, 2.5, 5, 10, 15, 20, and 25 μL glucose per tube). The method consists of adding 200 μL of 5% phenol to a glass tube containing 200 μL of the sample or standard curve point (in triplicate per sample). After careful mixing, 1 mL of sulfuric acid was added to each tube under agitation. After 20 min of reaction, samples were measured using a spectrophotometer (490 nm).

#### ASP analysis

Aliquots of 0.95 mL of each biofilm suspension were centrifuged (13,000 × *g*, for 10 min at 4°C). The supernatant of each tube was carefully removed and discarded. The pellets were then dried in a desiccator for 1 week. The pellets were weighed and 300 µL of 1 N NaOH per 1 mg of the dry weight was added. The pellets with 1 N NaOH were incubated for 2 h at 37°C and then centrifuged at 13,000 × *g* for 10 min. The supernatants were carefully collected with a pipette and transferred to new microcentrifuge tubes, preserving just the pellet. Once more, the same volume of 1 N NaOH as previously was added to the tubes containing the pellets, and the same steps as above were repeated for the extraction of ASP. After incubation, samples were centrifuged (13,000 × *g* for 10 min) and the supernatants were carefully collected and added to the previously collected supernatant. For the third extraction, the same steps as above were repeated, but this time, the samples were not incubated for 2 h before centrifugation. After three extractions, three volumes of cold 95% ethanol were added to each sample. The samples were then stocked at −20°C for 18 h for precipitation of ASP. After precipitation of ASP, the tubes were centrifuged (9,500 × *g* for 20 min at 4°C), and the supernatants were discarded. Each resulting pellet was washed three times with ice-cold 75% ethanol and air-dried, following the procedures performed for WSP samples. The pellets were resuspended in the same total volume of the original extraction with 1 N NaOH. Finally, the samples were ready for quantification of total carbohydrates using the phenol–sulfuric acid method as described for WSP analysis.

#### eDNA analysis

Aliquots of 0.65 mL of the supernatant of biofilm suspensions were mixed with an equal volume of phenol:chloroform:isoamyl alcohol (25:24:1) and once with chloroform:isoamyl alcohol (24:1) for eDNA extraction. The aqueous phase of each sample was mixed with three volumes of isopropanol and 1/10 volume of 3 M sodium acetate (pH 5.2) and stored at −20°C for 18 h. The eDNA precipitated with isopropanol was collected by centrifugation (13,000 × *g* for 20 min at 4°C) and washed three times with ice-cold 70% ethanol, air-dried, and then dissolved in 10 μL of TE buffer (Tris HCl/1 mM EDTA, pH 8.0). The amount of eDNA was determined using a spectrophotometer with light length of 260 nm.

#### Variable-pressure scanning electron microscopy (VPSEM) protocol

After 48 h of biofilm formation, the samples were transferred directly to the VPSEM (Zeiss EVO 50; Carl Zeiss Microscopy, LLC, Thornwood, NY, USA) chamber and imaged at 100 Pa. VPSEM images were captured at working distances of 6.5 mm and 7.0 mm and field widths of 10 µm and 20 µm [28].

#### Confocal scanning laser microscopy (CSLM)

The biofilm morphology was determined by CSLM, using a Leica TCS SP5 microscope (Leica Lasertechnik, Heidelberg, Germany) with an HCX APOL U-V-I 40X/0.8-numerical-aperture water immersion objective. The biofilms were stained with a live/dead viability kit (Molecular Probes; Invitrogen, Eugene, OR, USA). The stain was prepared by diluting 1.5 μL of SYTO 9 and 1.5 μL of propidium iodide in 1.0 mL of sterile 1% phosphate-buffered solution (pH 7.4) [29]. The plates were incubated at room temperature in the dark for 15 min and examined under a CSLM. The biovolume (μm^3^/μm^2^) and maximum thickness (μm) [30] of the biofilms were quantified using COMSTAT2 (http://www.comstat.dk), and the images were rendered in Amira software (Mercury Computer Systems, Chelmsford, MA, USA).

### Statistical analyses

All the experiments were repeated on three separate occasions, with four replicates (n = 12). Data were analyzed by one-way analysis of variance (ANOVA) with Tukey’s post-hoc test (*α* = 0.05). A Pearson’s correlation test (*r*) was applied to check correlations between the different ECM components. Correlation was considered significant at the 0.05 level (two-tailed). All the tests were performed using IBM SPSS Statistics 19 (IBM Corp., Armonk, NY, USA).

## Results

The quantitative data on population, dry weight, protein, and ECM components are displayed in Table 1. The population data demonstrated significant differences between WT and Δ/Δ efg1 and between Δ/Δ tec1 and Δ/Δ efg1 (*p* < 0.001), while no statistical differences were observed between WT and Δ/Δ tec1 strains. The dry weight (total biomass) of the WT strain was higher than that of the mutant strains (*p* < 0.005). The proteins were significantly higher (*p* < 0.05) in the Δ/Δ efg1 mutant strain. The ASP data showed significant differences for all of the strains (*p* = 0.000), with the WT strain possessing the highest amount of this ECM component (mean ± SD: 74.5 ± 22.0 µg), followed by Δ/Δ tec1 (44.0 ± 24.1 µg) and Δ/Δ efg1 (14.7 ± 5.0 µg). However, the other parameters, eDNA, WSP and matrix proteins, showed no significant differences among the strains (*p* > 0.05) (Table 1).

**Table 1.** Biofilm and extracellular matrix (ECM) components of *Candida albicans* biofilms.^a^

Biofilm components				ECM components			
Strain	Log_10_ cfu mL^−1^	Dry weight (mg)	Protein (μg)	ASP (μg)	WSP (μg)	eDNA (μg)	Matrix protein (μg)
*C. albicans* SN425 (WT)	6.89 ± 0.07a	24.5 ± 3.7a	47.8 ± 1.5a	74.5 ± 22.0a	47.7 ± 13.4a	20.1 ± 7.1a	13.8 ± 9.8a
*C. albicans* CJN2302 (Δ/Δ efg1)	7.16 ± 0.17b	18.4 ± 2.8b	50.3 ± 2.3b	14.7 ± 5.0b	52.2 ± 7.8a	27.4 ± 7.6a	20.4 ± 11.3a
*C. albicans* CJN2330 (Δ/Δ tec1)	6.83 ± 0.18a	18.5 ± 3.0b	46.5 ± 1.4a	44.0 ± 24.1c	46.1 ± 11.3a	34.2 ± 14.4a	17.4 ± 4.3a

^a^Data are shown as mean ± SD of colony count (log_10_ cfu mL^−1^), dry weight (mg), total protein (µg), alkali-soluble polysaccharide (ASP) (µg), water-soluble polysaccharide (WSP) (µg), extracellular DNA (eDNA) (µg), and matrix protein (µg) for *C. albicans* SN425 [wild-type (WT)], *C. albicans* CJN2302 (Δ/Δ efg1), and *C. albicans* CJN2330 (Δ/Δ tec1).

Comparisons by one-way ANOVA and Tukey’s post-hoc test: means followed by the same letter in a column are not significantly different from each other.

Pearson’s correlation was applied to compare the ECM components with each other. This analysis showed a significant correlation (*p* < 0.05) between the ASP and protein content in the WT strain (*r *= 0.666) as well as between the protein and matrix protein (*r = *0.670) (Table 2). The population of Δ/Δ efg1 significantly correlated with the protein content of its ECM (*r *= 0.734) (Table 3). The Δ/Δ tec1 mutant strain showed a significant correlation between the ASP content and the eDNA in its ECM (*r *= 0.678) (Table 4).

**Table 2.** Pearson’s correlation (*r*) of colony count (log_10_ cfu mL^−1^) and extracellular matrix (ECM) components for the wild-type (WT) strain (SN425).

WT strain (SN425)		Log_10_ cfu mL^−1^	ASP	WSP	Total protein	Matrix protein	eDNA
Log_10_ cfu mL^−1^	Pearson’s *r*	1	0.372	0.262	0.173	0.017	−0.167
	Sig. (2-tailed)		0.234	0.411	0.590	0.957	0.605
	*N*		12	12	12	12	12
ASP	Pearson’s *r*		1	0.184	**0**.666*	0.361	0.273
	Sig. (2-tailed)			0.567	**0**.**018**	0.249	0.391
	*N*			12	**12**	12	12
WSP	Pearson’s *r*			1	0.065	0.017	−0.326
	Sig. (2-tailed)				0.841	0.959	0.301
	*N*				12	12	12
Protein	Pearson’s *r*				1	**0**.670*	0.376
	Sig. (2-tailed)					**0**.**017**	0.229
	*N*					**12**	12
Matrix protein	Pearson’s *r*					1	−0.032
	Sig. (2-tailed)						0.921
	*N*						12
eDNA	Pearson’s *r*						1
	Sig. (2-tailed)						
	*N*						

ASP, alkali-soluble polysaccharide; WSP, water-soluble polysaccharide; eDNA, extracellular DNA; Sig., significance.

*Correlation is significant at the 0.05 level (two-tailed).

**Table 3.** Pearson’s correlation (*r*) of colony count (log_10_ cfu mL^−1^) and extracellular matrix (ECM) components for the Δ/Δ efg1 mutant strain (CJN2302).

Δ/Δ efg1 strain		Log_10_ cfu mL^−1^	ASP	WSP	Total protein	Matrix protein	eDNA
Log_10_ cfu mL^−1^	Pearson’s *r*	1	0.246	0.057	**0**.734*	−0.286	−0.315
	Sig. (2-tailed)		0.442	0.861	**0**.**010**	0.367	0.318
	*N*		12	12	**12**	12	12
ASP	Pearson’s *r*		1	0.477	−0.133	0.252	0.290
	Sig. (2-tailed)			0.117	0.696	0.429	0.361
	*N*			12	12	12	12
WSP	Pearson’s *r*			1	0.236	−0.160	0.080
	Sig. (2-tailed)				0.485	0.619	0.805
	*N*				12	12	12
Protein	Pearson’s *r*				1	−0.205	0.538
	Sig. (2-tailed)					0.545	0.071
	*N*					12	12
Matrix protein	Pearson’s *r*					1	−0.247
	Sig. (2-tailed)						0.463
	*N*						12
eDNA	Pearson’s *r*						1
	Sig. (2-tailed)						
	*N*						

ASP, alkali-soluble polysaccharide; WSP, water-soluble polysaccharide; eDNA, extracellular DNA; Sig., significance.

*Correlation is significant at the 0.05 level (two-tailed).

**Table 4.** Pearson’s correlation (*r*) of colony count (log_10_ cfu mL^−1^) and extracellular matrix (ECM) components for the Δ/Δ tec1 mutant strain (CJN2330).

Δ/Δ tec1 strain		Log_10_ cfu mL^−1^	ASP	WSP	Total protein	Matrix protein	eDNA
Log_10_ cfu mL^−1^	Pearson’s *r*	1	−0.080	0.171	−0.155	−0.171	−0.194
	Sig. (2-tailed)		0.804	0.596	0.630	0.595	0.567
	*N*		12	12	12	12	12
ASP	Pearson’s *r*		1	0.285	0.106	0.190	**0**.678*
	Sig. (2-tailed)			0.369	0.743	0.554	**0**.**022**
	*N*			12	12	12	**12**
WSP	Pearson’s *r*			1	−0.002	−0.535	0.219
	Sig. (2-tailed)				0.995	0.073	0.518
	*N*				12	12	12
Protein	Pearson’s *r*				1	0.000	0.098
	Sig. (2-tailed)					1.000	0.775
	*N*					12	12
Matrix protein	Pearson’s *r*					1	0.322
	Sig. (2-tailed)						0.334
	*N*						12
eDNA	Pearson’s *r*						1
	Sig. (2-tailed)						
	*N*						

ASP, alkali-soluble polysaccharide; WSP, water-soluble polysaccharide; eDNA, extracellular DNA; Sig., significance.

*Correlation is significant at the 0.05 level (two-tailed).

**Figure 2.**
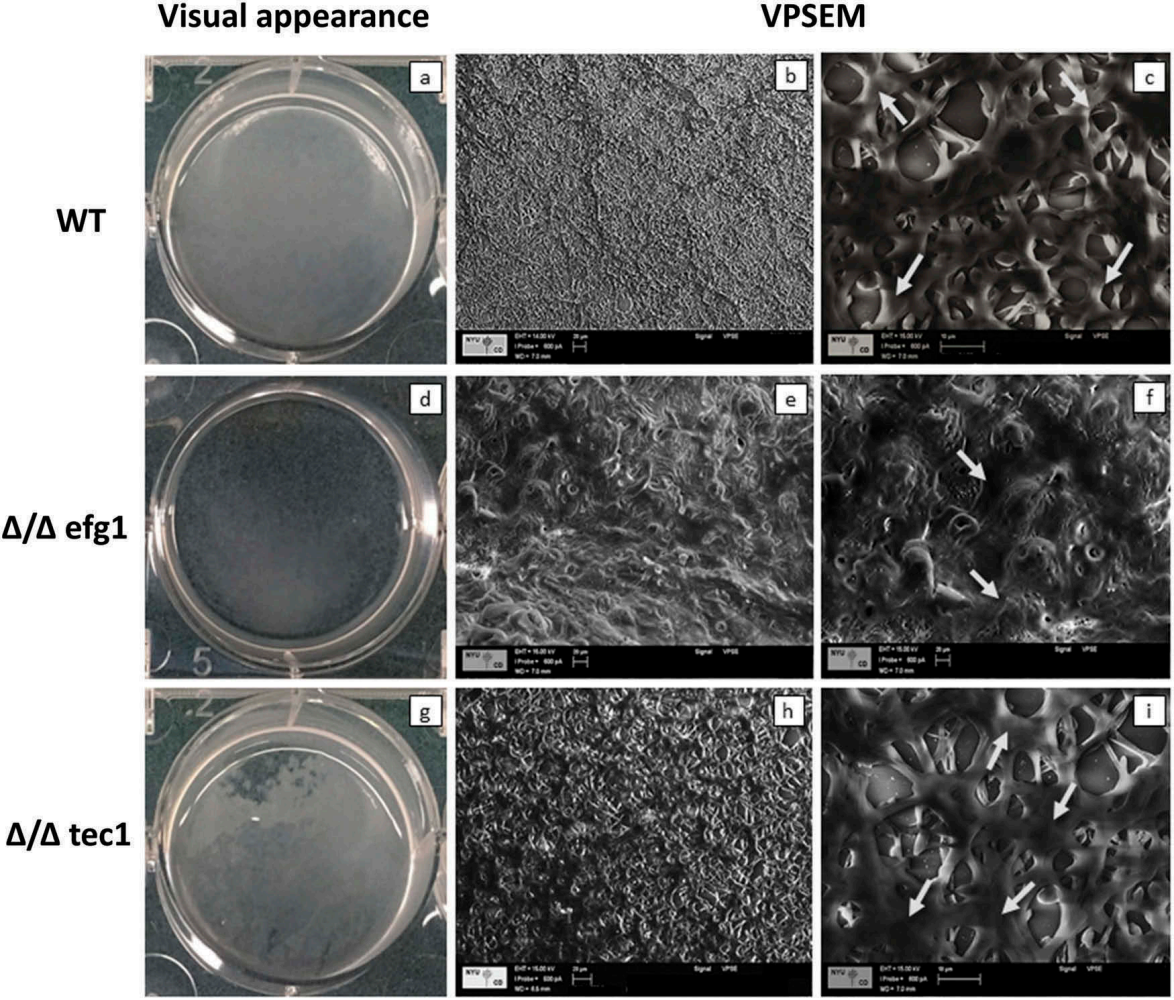
Visual macroscopic appearance and overall *Candida albicans* biofilm structure by variable-pressure scanning electron microscopy (VPSEM). The wild-type (WT) strain presents a typical thick biofilm architecture in visual appearance (a) with the presence of abundant extracellular matrix (ECM) (b, c), exemplified by the arrows (c). The Δ/Δ efg1 mutant shows sparse thin biofilm growth patterns (d) and an ECM (e, f) morphologically distinct from the WT (b, c) and Δ/Δ tec1 strains (h, i). The Δ/Δ tec1 mutant presents defects in visual appearance (g) compared to the WT strain (a); however, its ECM is morphologically similar to the WT (h, i, see arrows).

The WT strain presented a typical thick biofilm architecture in visual appearance (Figure 2(a)) with the presence of abundant ECM (Figure 2(b,c)), as observed by VPSEM. In contrast, the Δ/Δ efg1 mutant showed sparse thin biofilm growth patterns (Figure 2(d)) and morphologically distinct ECM (Figure 2(e,f)) compared with the WT (Figure 2(b,c)) and Δ/Δ tec1 strains (Figure 2(h,i)). The Δ/Δ tec1 mutant presented defects in visual appearance (Figure 2(g)) compared to the WT strain (Figure 2(a)); however, its ECM was morphologically similar to the WT (Figure 2(h,i)).

**Figure 3.**
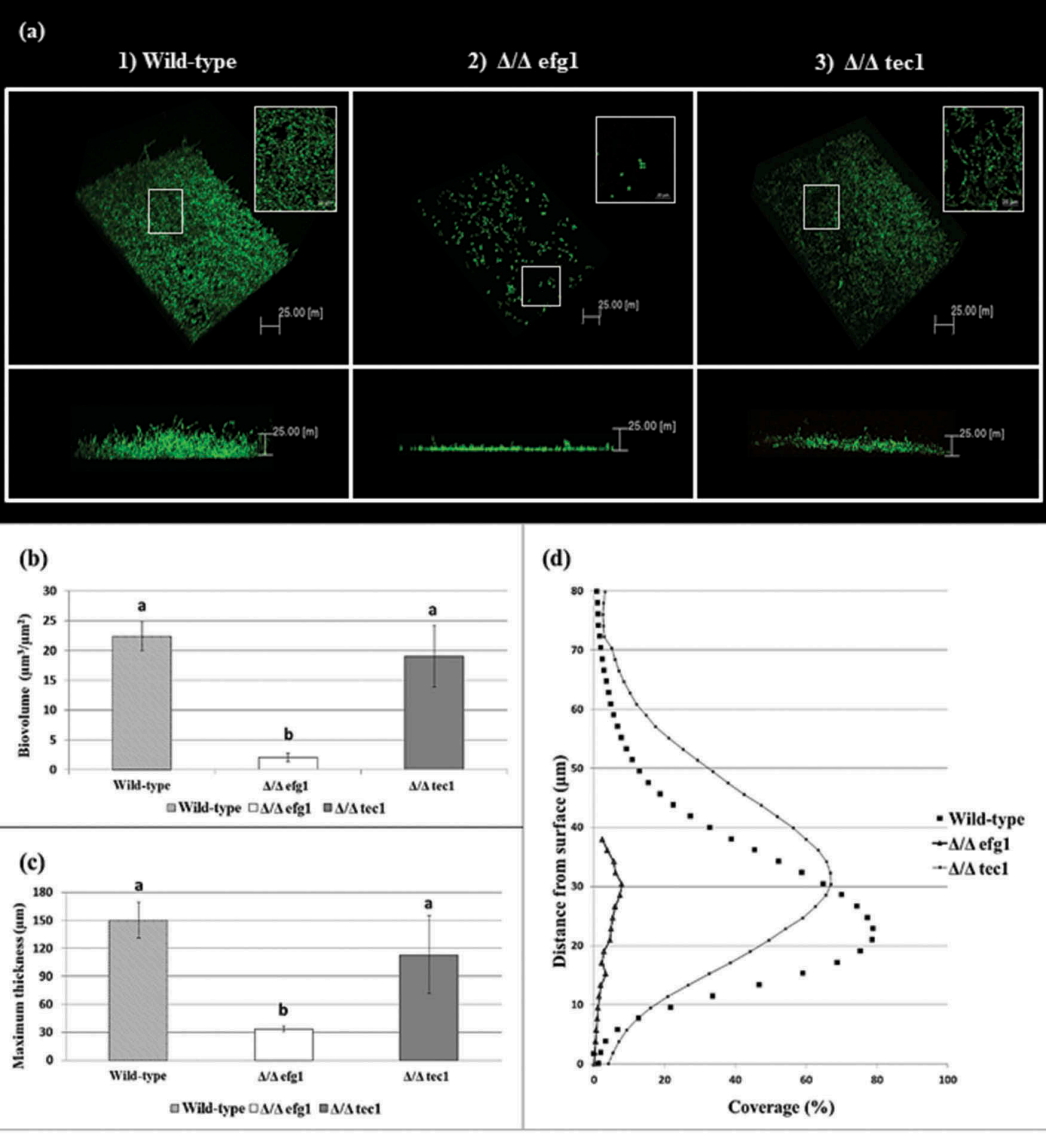
*Candida albicans* biofilm structures and corresponding quantitative data from confocal scanning laser microscopy assays. (a) Representative three-dimensional and orthogonal images of the structural organization of the 48 h biofilms of *C. albicans* wild-type (WT) (1) and mutant strains: Δ/Δ efg1 (2) and Δ/Δ tec1 (3) (green color denotes labeling with SYTO9 for live yeast cells). Mean and SD of biovolume (b) and average biofilm thickness (c) of *C. albicans* WT and mutant strains (Δ/Δ efg1 and Δ/Δ tec1) determined by COMSTAT2 analysis. The average biovolume and biofilm thickness were calculated from five independent samples from each strain. Values followed by the same letter are not significantly different (*p* > 0.05), as determined by an analysis of variance for all pairs using Tukey’s test. The profile of the distribution of *C. albicans* WT and mutant strains (Δ/Δ efg1 and Δ/Δ tec1) in the biofilms is represented in (d).

CSLM representative images of the 48 h biofilms of *C. albicans* strains are shown in Figure 3. Multidimensional imaging of live (green) cells can be observed at different depths of the biofilms. In addition, a negligible amount of dead (red-stained) cells was observed (not shown in Figure 3). The images show that the biofilm formed by the WT strain (Figure 3(a-1)) has an elevated number of hyphae, in contrast to the biofilm formed by the Δ/Δ efg1 mutant strain, which did not exhibit hyphae (Figure 3(a-2)). The biofilm formed by the Δ/Δ tec1 mutant strain (Figure 3(a-3)) exhibited hyphae, but in a smaller quantity than the WT strain (Figure 3(a-1)). The orthogonal view of the biofilms showed that the biofilms of mutant strains Δ/Δ efg1 (Figure 3(a-2)) and Δ/Δ tec1 (Figure 3(a-3)) were thinner than that of the WT (Figure 3(a-1)); in particular, the Δ/Δ efg1 had the thinnest structure, as confirmed by the biovolume (Figure 3(b)) and maximum thickness of the biofilms (Figure 3(c)). The profiles of the distribution of *C. albicans* WT and mutant strains in the 48-h-old biofilms are shown in Figure 3(d), where it can be observed that the biofilm profiles of the WT and Δ/Δ tec1 strains are similar, while the Δ/Δ efg1 mutant shows a very low percentage of coverage.

## Discussion

The biofilm architecture contributes to the maintenance of stable interactions between cells, and between cell surfaces and the environment [31]. Furthermore, it protects against phagocytic cells and works as a scaffold for preserving biofilm integrity by limiting the diffusion of noxious substances into the biofilm [32]. Although biofilm resistance is multifactorial [33], the protection exerted by the ECM is a key factor in the high levels of antifungal drug resistance displayed by *C. albicans* biofilms [13,14,34]. It has been demonstrated that transcriptional regulatory genes in *C. albicans*, including TEC1 and EFG1, regulate biofilm formation [23,35,36]. Thus, understanding how these transcriptional regulatory genes influence the ECM composition is paramount to better preventing or impairing biofilm formation.

The present study demonstrated that ASPs are major components of the ECM of the WT strain, and that these components are significantly reduced in the mutant strains with biofilm formation deficiency (Δ/Δ efg1 and Δ/Δ tec1), with Δ/Δ efg1 being the strain that possessed the smallest quantities of these components. A significant correlation (*p* < 0.05) between the protein and the ASP content in the ECM of the WT strain (*r *= 0.666) was present, so the production of ASP in this strain may be influenced by the proteins and *vice versa* (Table 2). It appears that filamentous cells build up ECM that is richer in ASPs than in non-filamentous cells. Early studies showed a correlation between ECM and levels of resistance against fluconazole and amphotericin B [37,38]. An ECM component with a role in antifungal drug resistance is β-1,3-glucan (an ASP), which acts through a mechanism of drug sequestration.

Delivery of β-1,3-glucan to the ECM is controlled by a glucan-modifying pathway composed of Bgl2p and Phr1p (glycosyltransferases) and Xog1p (glucanase) [39]. It has been shown that the deletion of any of the genes encoding these proteins resulted in at least 10-fold reduction in the matrix β-1,3-glucan content and formed more vulnerable biofilms, which were easily disrupted [39]. Moreover, biofilms formed by these mutants showed less ability to sequester fluconazole and higher susceptibility to this drug [39]. In addition to drug sequestration, it has been demonstrated that the production of β-1,3-glucan by *C. albicans* biofilms hinders the production of reactive oxygen species by neutrophils and protects the cells in the biofilm from neutrophil killing [40]. β-1,3-glucan binds to fluconazole, preventing this drug from reaching its cellular targets [13,39,41,42]. A similar effect was detected for amphotericin B [18] and for other classes of antifungal agents [41]. It has been demonstrated that EFG1 intermediates tolerance of *C. albicans* to azoles (i.e. fluconazole, ketoconazole, and itraconazole) and polyenes, including amphotericin B [43]. Moreover, a mutant Δ/Δ efg1 *C. albicans* strain showed higher susceptibility to these drugs, including miconazole and caspofungin [43,44]. Thus, the ASP of the ECM has a potential role in the tolerance to antifungals and biofilm structure, since it is accumulated much more in the ECM of the WT strain than in the more susceptible mutant Δ/Δ efg1.

Another ECM component with a role in antifungal drug resistance is eDNA. This component is a key matrix component of fungal and bacterial biofilms that enables adhesion to distinct surfaces and binds with other biopolymers, giving biofilms structural integrity and stability [34,45,46]. The addition of DNase increases the susceptibility of mature *C. albicans* biofilms to some antifungal agents [47]. The present study found a significant correlation between the eDNA and ASP content in the ECM of the Δ/Δ tec1 mutant strain (*r *= 0.678) (Table 4), indicating that the production of these components occurs together in this strain. Although the precise mechanism by which eDNA is released and contributes to drug resistance remains unclear [34], it has been suggested that eDNA may be released during hyphal growth [48]. However, the present study shows that, independent of the presence of hyphae, all the biofilms produced similar quantities of eDNA, in contrast to findings for ASP, where filamentous cells produced more ASP that non-filamentous cells. Therefore, the mutant strain Δ/Δ efg1 does not form hyphae but can produce eDNA and this indicates that eDNA production is not necessarily related to hyphal growth.

The Δ/Δ efg1 population (log_10_ cfu mL^−1^) was higher than that of WT and Δ/Δ tec1 cells. The smaller size and unicellularity can be considered reasons to explain differences in fungal population observed between mutant Δ/Δ efg1 and WT strains [49]. Moreover, cells lacking EFG1 genes showed increased colonization of the gastrointestinal tract of mice [50]. As the Δ/Δ efg1 mutant strain did not form hyphae, the smaller size of its cells may have supported the higher number of viable cells for colony counting. In contrast, the Δ/Δ tec1 and WT strains had similar populations, which were both statistically smaller than the Δ/Δ efg1 mutant strain. This result was probably due to these strains presenting hyphae. However, the difference in population between all the strains was less than 1 log, meaning that this result may not be biologically significant. On the other hand, the biomass of the mutant strains (Δ/Δ efg1 and Δ/Δ tec1) was lower than the biomass of the WT strain, confirming that these mutants have a defective capability to form biofilms. The biovolume of the mutant strains was also lower than that of the WT (Figure 3).

The quantitative analysis of proteins showed that the mutant strain Δ/Δ efg1 had a higher content of proteins than the WT and Δ/Δ tec1 mutant strains, which makes sense because the log_10_ cfu mL^−1^ significantly influenced the protein in the biofilm of this strain (*r *= 0.734) (Table 3). Pearson’s correlation test applied to the current data showed a significant (*p* < 0.005) correlation between the protein and the matrix proteins (*r *= 0.670) in the WT strain (Table 2). Proteins make up a large portion of the biomass in many microbial biofilms [31,51,52], having been shown to comprise more than half of the *C. albicans* ECM by weight [16]. A comparison of the matrix proteome of *C. albicans* and total matrix proteins identified several similarities between them, including a large amount of proteins involved in carbohydrate and amino acid metabolism [53,54]. The known function of most proteins is related to metabolism [16], suggesting that the ECM may ‘function as an external digestive structure that disrupts extracellular biopolymers as an energy source’ [16]. Thus, the higher amounts of protein related to the higher population values observed in the Δ/Δ efg1 may represent these strains making an extra effort to obtain energy to arrange as a biofilm.

Evaluation of intact biofilm architecture by VPSEM and CSLM provides valuable information on cells and ECM spatial organization. VPSEM preserves the ECM since it does not require sample dehydration processes and high chamber vacuum [28]. The images showed that while the WT strain presented a typical bulky biofilm architecture encased by ECM material (Figure 2(b,c)), the Δ/Δ efg1 mutant strain was morphologically distinct from the WT (Figure 2(e,f)). On the other hand, the Δ/Δ tec1 mutant formed a biofilm with ECM comparable to the WT (Figure 2(h,i)). Thus, the similarity between the ECM produced by the WT and Δ/Δ tec1 mutant strain can be related to the growth of pseudohyphae and hyphae, which goes along with the production of ECM [55].

Moreover, the CLSM images corroborated the results obtained with VPSEM, showing that the WT reference strain formed a thick and bulky biofilm on the surface of the polystyrene plate (Figure 3(a-1)). However, the mutant strains tested showed defects in biofilm formation, especially Δ/Δ efg1, which was severely defective (Figure 3(a-2)), while the Δ/Δ tec1 mutant had less pronounced defects (Figure 3(a-3)). These results agreed with a study that analyzed the same strains [23]. The measurements of the biovolume and maximum biofilm thickness obtained by COMSTAT2 confirmed these results, showing that the Δ/Δ efg1 mutant strain had the lowest biovolume and maximum thickness (*p* > 0.05) of the studied strains. The biofilm profile of this strain was markedly different from that of the other strains, showing a small percentage of coverage area (Figure 3(d)). In addition, previous studies showed that Δ/Δ efg1 mutant strains have impaired hyphae growth under many conditions [19,21]. Contrary to a previous report that found that biofilm produced by the tec1 null mutant (Δ/Δ tec1) strain was composed exclusively of yeast cells [17], it was observed in the present study that Δ/Δ tec1 is a defective mutant that exhibits hyphae; however, its biofilm had a defective visual appearance and a smaller quantity of hyphae compared to the WT strain (Figure 3(a-3)). Despite the visual and microscopic differences between the WT and Δ/Δ tec1 strains, they had a comparable biovolume and maximum biofilm thickness (*p* > 0.05); in addition, the biofilm percentage coverage profile of these strains was similar (Figure 3(d)).

In conclusion, this study characterized the ECM of *C. albicans* WT and mutant strains derived from it. Despite the Δ/Δ efg1 mutant strain presenting severe biofilm defects, its cells grew more in number than the WT and Δ/Δ tec1 mutant strains and it matched the higher quantity of proteins in the biofilm. The amounts of eDNA, WSPs, and matrix soluble proteins were similar between the strains, but the eDNA correlated with the ASP content in the ECM of the Δ/Δ tec1 strain. On the other hand, the ASP content was significantly higher in the WT strain than in the mutant strains, which indicates that ASP production may be linked to *C. albicans* cell filamentous morphology.

## Supplementary Material

Supplemental_material_revised__1_.docx
