# Supplementary material for: Inactivation of genes TEC1 and EFG1 in Candida albicans influences extracellular matrix composition and biofilm morphology
Source: J Oral Microbiol. 2017 Oct 17;9(1):1385372. doi: 10.1080/20002297.2017.1385372 (PMC5646609; doi:10.1080/20002297.2017.1385372)
Supplement: Supplemental_material_revised__1_.docx [file ZJOM_A_1385372_SM8100.docx]

**Figure S1**: Growth curves of *C. albicans* strains SN 425 (WT), CJN 2302 (Δ/Δ efg1) and CJN 2330 (Δ/Δ tec1). Planktonic cultures were performed in YNB medium supplemented with 100 mM of glucose and incubated at 37º C. The optical density (OD at 540 nm) and the population (Log_10_ CFU mL^-1^) were determined over time.

**Table S1.** Biofilm and ECM components of *C. albicans* biofilms normalized by the dry weight (DW). Mean and standard deviations of dry weight (mg), Log_10_ (CFU mL^-1^)/DW, eDNA/DW (µg), WSP/DW (µg), ASP/DW (µg), matrix protein/DW (µg) and protein/DW (µg) for *C. albicans* SN 425 (wild-type), *C. albicans* CJN 2302 (Δ/Δ efg1) and *C. albicans* CJN 2330 (Δ/Δ tec1).

| Biofilm Components | | | | | | | |  | ECM components | | | | | | | |
| --- | --- | --- | --- | --- | --- | --- | --- | --- | --- | --- | --- | --- | --- | --- | --- | --- |
| Strains |  | | |  | CFU mL^-1^  Log_10_/DW |  | Protein/DW (μg/mg) |  | ASP/DW (μg/mg) |  | eDNA/DW (μg/mg) |  | WSP/DW (μg/mg) |  | Matrix protein/DW (μg/mg) |  |
|  | Dry Weight DW (mg) | | |  |  |  |  |  |  |  |  |  |  |  |  |  |
| *C. albicans* SN 425 (WT) | | 21.5 | A | | 0.32 | A | 2.2 | A | 3.5 | A | 1.0 | A | 2.2 | A | 0.6 | A |
|  | | (2.3) |  | | (0.03) |  | (0.2) |  | (1.1) |  | (0.4) |  | (0.7) |  | (0.4) |  |
| *C. albicans* CJN 2302 (Δ/Δ efg1) | | 18.3 | B | | 0.39 | B | 2.8 | B | 0.8 | B | 1.5 | A | 2.8 | A | 1.0 | A |
|  | | (2.6) |  | | (0.06) |  | (0.4) |  | (0.2) |  | (0.5) |  | (0.2) |  | (0.6) |  |
| *C. albicans* CJN 2330 (Δ/Δ tec1) | | 18.6 | B | | 0.37 | A | 2.5 | A | 2.3 | C | 1.6 | A | 2.4 | A | 1.0 | A |
|  | | (2.7) |  | | (0.05) |  | (0.4) |  | (1.2) |  | (0.5) |  | (0.4) |  | (0.3) |  |
|  | |  |  | |  |  |  |  |  |  |  |  |  |  |  |  |

Comparisons by one-way ANOVA and Tukey post-hoc test: means followed by the same letter in column are not significantly different from each other.
